# Molecular Epidemiology of Ovine Papillomavirus Infections Among Sheep in Southern Italy

**DOI:** 10.3389/fvets.2021.790392

**Published:** 2021-11-22

**Authors:** Francesca De Falco, Anna Cutarelli, Nicola D'Alessio, Pellegrino Cerino, Cornel Catoi, Sante Roperto

**Affiliations:** ^1^Dipartimento di Medicina Veterinaria e Produzioni Animali, Università degli Studi di Napoli Federico II, Naples, Italy; ^2^Istituto Zooprofilattico Sperimentale del Mezzogiorno, Portici, Italy; ^3^Department of Pathology, Faculty of Veterinary Medicine, University of Agricultural Sciences and Veterinary Medicine, Cluj-Napoca, Romania

**Keywords:** droplet digital polymerase chain reaction, liquid biopsy, molecular epidemiology, ovine papillomavirus, real-time quantitative PCR

## Abstract

Ovine papillomaviruses (OaPVs) were detected and quantified, for the first time, using droplet digital polymerase chain reaction (ddPCR) and real-time quantitative PCR (qPCR) *via* blood samples of 165 clinically healthy sheep. OaPV DNA was detected in 126 blood samples (~76.4%). DdPCR detected OaPV DNA in 124 samples; in only two additional samples positive for real-time qPCR, ddPCR failed to detect the presence of any OaPVs. In 70 of the positive samples (~55.6%), a single OaPV infection was observed, 12 of which were caused by OaPV1 (~17.1%) and 14 by OaPV2 (20%). OaPV3 was responsible for 19 single infections (~27.1%), and OaPV4 for 25 single infections (~35.7%). Multiple OaPV coinfections were observed in 56 (~44.4%) positive samples. OaPV coinfections caused by two genotypes were observed in 31 positive samples (~55.4%), with dual OaPV3/OaPV4 infection being the most prevalent as seen in 11 blood samples. In addition, five OaPV1/OaPV4, four OaPV1/OaPV2, four OaPV2/OaPV3, four OaPV1/OaPV3, and three OaPV2/OaPV4 dual coinfections were also detected. OaPV coinfections by triple and quadruple genotypes were detected in 24 (~42.8%) and only one (~1.8%) of coinfected blood samples, respectively. Multiple infections caused by OaPV1/OaPV3/OaPV4 genotypes were the most prevalent, as observed in 12 (50%) blood samples harboring triple OaPV infections. This study showed that ddPCR is the most sensitive and accurate assay for OaPV detection and quantification thus outperforming real-time qPCR in terms of sensitivity and specificity. Therefore, ddPCR may represent the molecular diagnostic tool of choice, ultimately providing useful insights into OaPV molecular epidemiology and field surveillance.

## Introduction

Papillomaviruses (PVs) are small, non-enveloped, double-stranded DNA viruses that infect mammals, reptiles, birds, and fish (1). In mammals, PV infections have been reported in wild and domestic, large, and small ruminants (2–7). At present, 29 genotypes of bovine papillomaviruses (BPVs) are known to infect large ruminants such as cattle and buffaloes (3, 5). In small ruminants, Capra hircus papillomavirus type 1 (ChPV1) and ChPV2 are the only two caprine genotypes responsible for PV-associated diseases in goats (1, 8). Species-specific PV infections are also known to occur in sheep. Ovine papillomaviruses (OaPVs) comprise four members, namely OaPV1, OaPV2, OaPV3 and OaPV4. OaPV1, OaPV2 and OaPV4 form OaPV species three within the genus *Delta*-papillomavirus, whereas OaPV3 belongs to the genus *Dyokappa-*papillomavirus^1^. OaPVs have been suggested to be associated with skin tumors (2, 9–13), as ultrastructural electron-dense particles showing papillomaviral features in symmetry and size have been observed in cutaneous papillomas and papillomatosis of sheep (2, 14). Furthermore, using cell- and bacteria-free inocula obtained from ovine warts, an experimental infection resulting in cutaneous proliferative lesions was transmitted to healthy sheep (2). It has been suggested that OaPVs may be involved in rumen papillomas of sheep (15). Although the complete genomes of OaPV1 and OaPV2 have been reported a long time ago^1^, their actual role in the molecular pathway involved in cutaneous and mucosal tumorigenesis of sheep remains to be elucidated, as their association with skin tumors has been poorly investigated in sheep (16). OaPV3 and OaPV4 have been recently identified in tumors of sheep from the Mediterranean region (Sardinia Island, Italy) (16, 17). It has been suggested that OaPV3 may represent a key factor in the pathway of ovine cutaneous squamous cell carcinoma (SCC), as OaPV3 DNA was detected in up to 65% of ovine SCCs (18). Furthermore, OaPV4, which appears to be most closely related to OaPV1, has been identified in sheep fibropapilloma (17). It has been shown that E6 and E7 are the major oncoproteins through which OaPV3 and OaPV4 immortalize primary sheep keratinocytes; however, only OaPV3 displays its transforming activity through both E6 and E7 oncoproteins (19). Ovine *Delta-*PVs share several biological properties with bovine *Delta*-PVs, such as cell tropism, as they can infect epithelial and mesenchymal cells (17). Similar to bovine *Delta*-PV, it has been suggested that the biological properties of ovine *Delta*-PV may be characterized by cross-species transmission. OaPV2 DNA sequences have been found in a sarcoid-like mass in the mouth of a pig (20).

Digital polymerase chain reaction (dPCR) is a new generation of PCR techniques that enables accurate absolute quantification of target molecules with high sensitivity. Droplet digital PCR (ddPCR) allows massive partitioning of DNA of the sample into millions of nanoliter-sized droplets that ideally contain either no particles or a single particle (21). Recently, ddPCR has been reported to detect and quantify bovine papillomaviruses BPVs in cattle, goats, and sheep (22–24). DdPCR has been shown to have higher accuracy than real-time quantitative PCR (qPCR). Therefore, ddPCR is currently the most accurate and sensitive method for measuring the abundance of nucleic acids of interest. DdPCR has demonstrated superior diagnostic performance than other available molecular techniques and is very useful in detecting low nucleic acid concentrations of oncogenic viruses, including PVs (25). Therefore, ddPCR technology is important in performing epidemiological investigations on the incidence ratio of PVs and their territorial prevalence.

This study aimed to investigate OaPV detection and quantification in the blood of apparently healthy sheep using ddPCR. In addition, the ddPCR assay data for OaPV detection and load quantification were compared to real-time quantitative PCR (qPCR) as qPCR is considered to be the standard, method with the highest sensitivity and specificity for detecting PVs DNA and cDNA (25).

## Materials and Methods

### Blood Samples and DNA Extraction

Blood samples from 165 healthy 1- to 10-year-old sheep living in regions of Southern Italy (Sarda breed from Sardinia, Lacaune and Bagnolese from Campania, Brianzola and Camusana from Calabria, Gentile di Lucania, Gentile di Puglia, and Sopravissana from Basilicata, Gentile di Puglia and Leccese from Apulia, and animals from the hybridization with local breeds) were collected from the jugular vein in vacutainers containing ethylenediaminetetraacetic acid (EDTA). Total DNA was extracted using a DNeasy Blood & Tissue Kit (Qiagen, Wilmington, DE, USA), according to the manufacturer's instructions.

### Positive Controls

The positive controls of OaPV1 and OaPV 2 were artificially created plasmids (vector: pUCIDT-AMP), containing 270 and 603 base pairs of the sequence of E5 and the major capsid protein, respectively (IDT, Integrated DNA Technologies, IA, USA). The positive control of OaPV3 was a plasmid (vector: pUC19) that contained the complete genome of OaPV3, and the positive control tissue for OaPV4 was a cutaneous fibropapillomatosis sample, both from the Department of Veterinary Medicine of Sassari University (kind gifts from Prof. A. Alberti).

### qPCR

Using the real-time qPCR assay, the online web interface from IDT^2^ primers and probes were designed. The amplicon length was set by the program to obtain 70–150 bp within the target regions. The primers and probes used for the detection of the four OaPV genotypes (OaPV1-2-3 and 4) are reported in Table 1. Primers and probes were ordered as a mix with a primer-to-probe ratio of 3.6. The qPCR reaction mixture was prepared by adding 7 μL of template (100 ng genomic DNA), 10 μL of 2X SsoAdvanced™ Universal Probes Supermix (Bio-Rad Laboratories, Hercules, CA, USA), 1 μL of target probe (FAM) /primer mix (final concentration of 900 nM of each primer and 250 nM of probe) in a total volume of 20 μl. DNA quality and concentration were assessed using a Nanodrop (Thermo Scientific, MA, USA). Four separate PCR reactions were performed using the CFX96 Real-Time System of the C1000 Touch^TM^ Thermal Cycler (Bio-Rad Laboratories, Hercules, CA, USA). The thermal cycling conditions were as follows: 50°C for 2 min, 95°C for 10 min, and 40 cycles of 95°C for 15 s and 58°C for 60 s. Each sample was analyzed in duplicate, and negative controls were included in all runs. Data acquisition and analysis were performed using the CFX Maestro™ (Bio-Rad Laboratories, Hercules, CA, USA) software. The same samples used as positive controls for ddPCR were also tested using qPCR.

**Table 1:** Primers and probes used for the detection of OaPVs in ddPCR and qPCR.

	**Forward 5^**′**^ 3^**′**^**	**Reverse 5^**′**^ 3^**′**^**	**Probe**	**Region**	**Size-bp**
OaPV1	CCTGATTCTATGACTGTAAGAGGC	CTCCCCACAGAAGTCCAAG	TGCAACAGCAGAGTCCCATCAGAAG FAM	E5 5′UTR/ORF E5	119
OaPV2	AGTTCCCGCTCTGATTTACC	ATGGCGGACGTATACTTGTTC	ATTGCCAGCAGTCTCCTCAGTCATTC FAM	Major capsid protein	134
OaPV3	AACTATGCAGGAATGTACGAGG	AGTTTCTCTGACAGGTTGCAC	TTGAGCTGGATGTGAGGTGTGTGAC FAM	E6	145
OaPV4	GGGTTCTATGGTGTCTGCTTAG	GCTCAAAATGGTCTACTGTTGC	CAGGAATGCTCTGTGCAGGGTATAGTG FAM	E6	102

### ddPCR

For ddPCR, Bio-Rad QX100 ddPCR System was used according to the manufacturer's instructions. The reaction was performed in a final volume of 22 μL containing 11 μL of ddPCR Supermix for Probes (2X; Bio-Rad), 0.9 μM primer, and 0.25 μM probe (Table 1) with 7 μL sample DNA corresponding to 100 ng. A black hole quencher was used in combination with FAM fluorescent dye reporters (Bio-Rad Laboratories, Hercules, CA, USA). The ddPCR mixture was placed into a 96-well PCR plate, and 7 μL of each sample was added to each well (Bio-Rad Laboratories, Hercules, CA, USA). The plate was transferred to an automated droplet generator (AutoDG, Bio-Rad Laboratories, Hercules, CA, USA). The AutoDG added 70 μL of droplet generation oil for the probe in every well, and each sample was partitioned into ~ 20,000 stable nano-droplets. The droplet emulsion (40 μL) was transferred into a new 96 well PCR plate and, then coated with a pierceable film heat sealed using a PX1 PCR Plate Sealer (Bio-Rad Laboratories, Hercules, CA, USA). PCR amplification was performed on a T100 Thermal Cycler (Bio-Rad Laboratories) with the following thermal profile: hold at 95°C for 10 min, 40 cycles of 94°C for 30 s, 58°C for 1 min, 1 cycle at 98°C for 10 min, and ending at 4°C. After amplification, the plate was loaded onto a droplet reader (Bio-Rad Laboratories, Hercules, CA, USA) and the droplets from each well of the plate were read automatically. A 96-well PCR plate was placed on the reader. Data were analyzed using the QuantaSoft analysis tool (Bio-Rad Laboratories, Hercules, CA, USA). Poisson statistics were used to calculate the absolute concentration of OaPV DNA in each sample (26). To discriminate between positive (blue) and negative (gray) droplets, a manual threshold line was used. There were also differences in the fluorescence amplitude range of the background (negative) droplets among the OaPV samples, that is, 4,000–8,000 for OaPV1; 3,000–6,000 for OaPV2; 4,000–10,000 for OaPV3; and 4,000–12,000 for OaPV4. Therefore, the ddPCR results could be directly converted into copies/μL in the initial samples simply by multiplying them by the total volume of the reaction mixture (22 μL) and then dividing that number by the volume of DNA sample added to the reaction mixture (7 μL) at the beginning of the assay. Each sample was analyzed in duplicate. Samples with very few positive droplets were re-analyzed to ensure that these low copy number samples were not due to cross-contamination.

### Limit of Detection (LoD) Determination

The four OaPV viral genes were detected using qPCR and ddPCR standard curves of the positive controls used in serial dilutions. A calibration curve of the positive sample dilutions (log10) was plotted against the PCR cycles. The linear range was determined by diluting the positive controls from 10^5^ to 10^−1^ copies/μL, detecting each dilution three times, taking the average value, and correlating the result with the theoretical value. In qPCR, the correlation of *R*^2^ > 0.98 was similar with the requirements of the test, and a Ct value of 40 was set as the minimum amount of viral detection assay. The lower detection limit obtained by ddPCR with values < 1 copies/μL indicated high sensitivity.

### Statistical Analysis

McNemar's Test for two Related Binomial Proportions (Conditional) was used to evaluate the agreement between the two tests performed on the same animals. To evaluate the actual difference in the prevalence of the four types of papillomavirus in the same animals, the Cochran-Armitage Test was performed. *P* < 0.05 was considered to be statistically significant.

## Results

**Figure 1:**
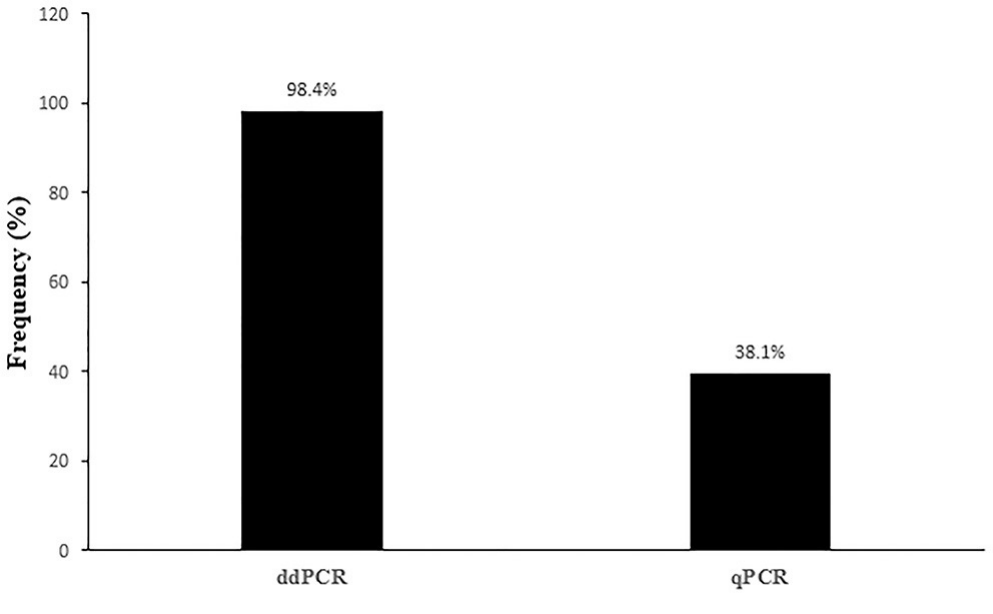
Percentages of positive samples containing OaPV DNA detected *via* ddPCR and qPCR methods.

**Figure 2:**
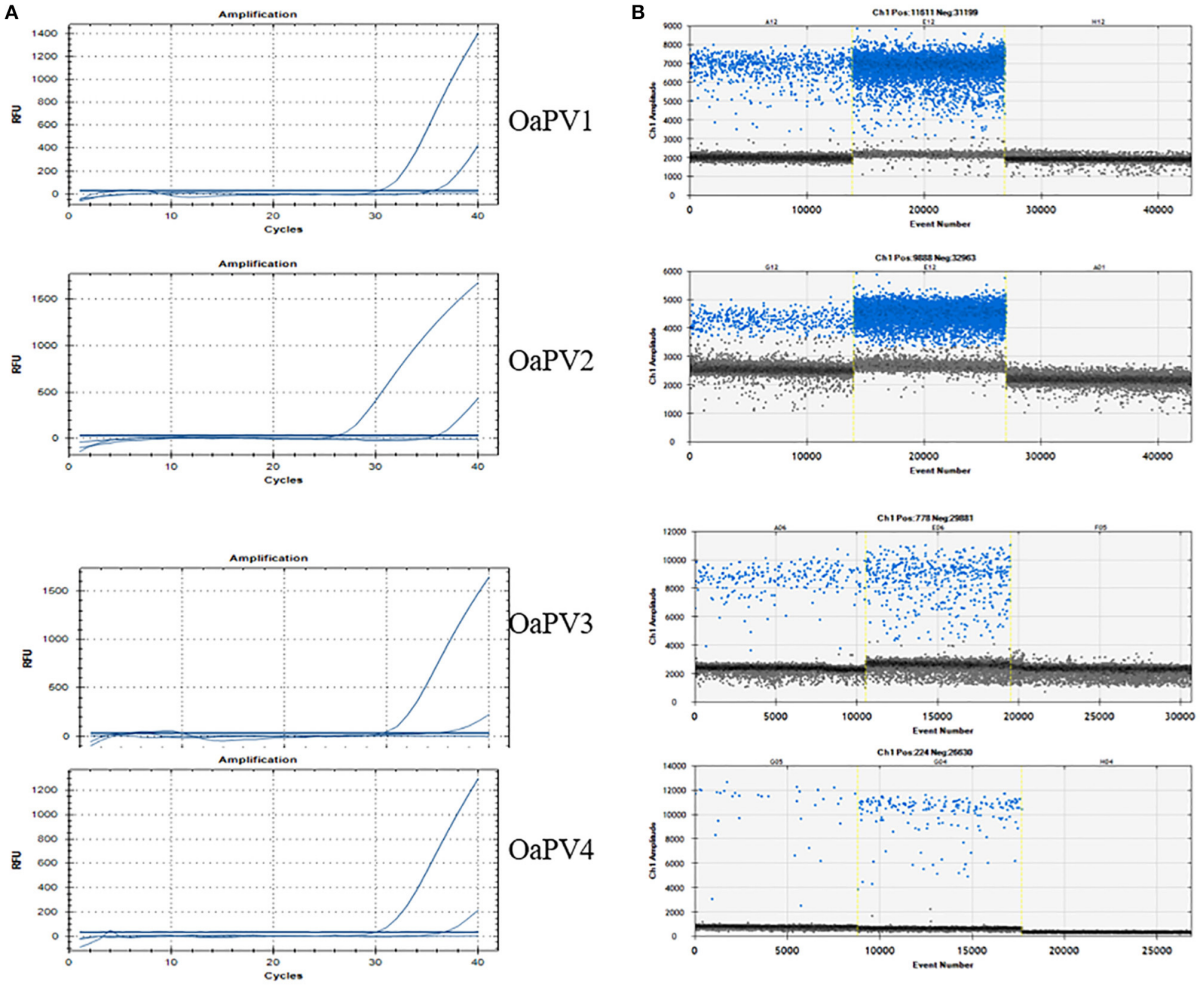
qPCR curves **(A)** and the relative rain plots of the ddPCR **(B)** for the four OaPVs. For all OaPVs one positive sample, the positive control, and one negative sample are shown.

In summary, OaPV DNA was found in 126 of the 165 blood samples examined (~76.4%) from healthy sheep using both ddPCR and real-time qPCR protocols; 39 sheep did not harbor any OaPV DNA. DdPCR detected OaPV DNA in 124 positive blood samples (~98.4.%) whereas real-time qPCR revealed OaPV DNA in 48 positive blood samples (~38.1%) (Figure 1), 46 of which were shown to harbor OaPV DNA using two methods. Differences between the two molecular protocols in detecting OaPV DNA were statistically significant, as the McNemar's test showed a *p* < 0.05. Table 2 details these results. Figure 2 shows the cycle threshold (Ct) for the qPCR results for both positive and negative samples. Data from qPCR were compared to those obtained *via* ddPCR performed on the same samples that correlated Ct and copy number obtained using qPCR and ddPCR, respectively (Supplemental Table S1).

**Table 2:** This table details results from 165 blood samples of healthy sheep.

		**ddPCR**		
		**+ ve**	**− ve**	**Total**
qPCR	+ ve	46	2	48
	− ve	80	37	117
Total		126	39	165

*OaPV DNA was detected in 126 blood samples. qPCR revealed OaPV DNA in 48 samples, 46 of which were also positive to ddPCR. Both methods failed to detect any OaPV DNA in 39 blood samples*.

**Figure 3:**
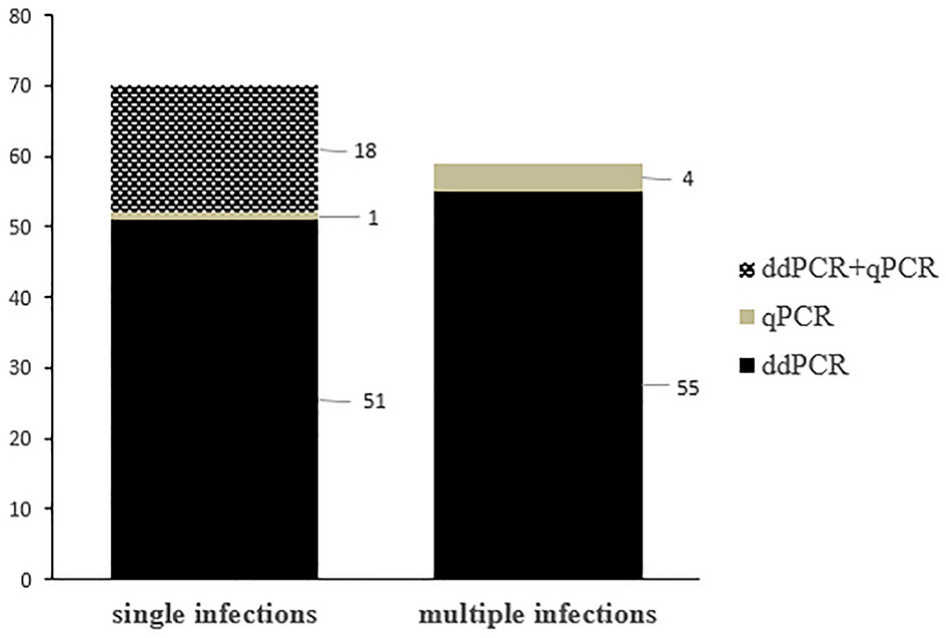
Graphical representation of single and multiple OaPV infections, as detected by ddPCR and qPCR.

**Figure 4:**
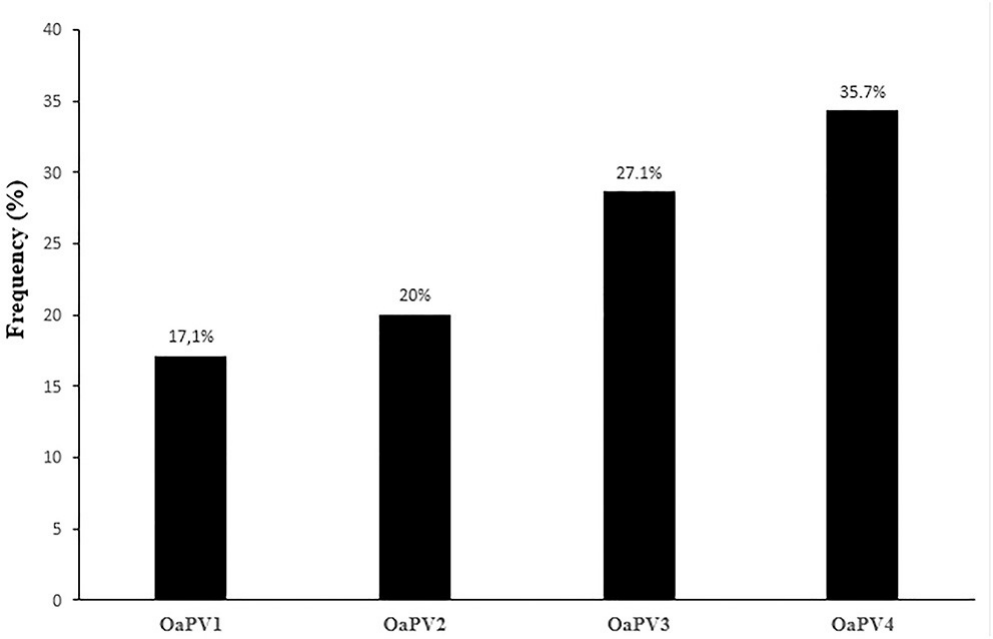
Detection rates of single OaPV DNA found in 70 samples positive for a single infection.

Single OaPV infection was observed in 70 positive samples (~ 55.6%) whereas multiple OaPV coinfections were observed in 56 positive samples (~ 44.4%). DdPCR detected single infections in 51 samples; 18 single infections were detected by both ddPCR and qPCR. In only one case, qPCR detected DNA of an OaPV genotype, causing a single infection that ddPCR did not detect (Figure 3). Overall, OaPV1 DNA was detected in 12 out of 70 single infections (~17.1%) and OaPV2 DNA in 14 (20%). OaPV3 and OaPV4 were responsible for 19 (~27.1%) and 25 (~35.7%) single infections, respectively (Figure 4). Differences in OaPV DNA detection were statistically significant as the Cochran-Armitage Test showed a *p* < 0.05. Both methods detected a greater number of positive samples to OaPV3 and OaPV4 than positive samples to OaPV1 and OaPV2.

OaPV double coinfections caused by the two genotypes were observed in 31 positive samples harboring multiple OaPV DNA (~ 55.4%). DdPCR detected 30 double infections, with OaPV3/OaPV4 genotype combination being the most prevalent infection, as observed in 11 blood samples. In addition, five coinfections composed of OaPV1/OaPV4, four OaPV1/OaPV2, four OaPV2/OaPV3, three OaPV1/OaPV3, and three OaPV2/OaPV4 were also detected. qPCR detected only four dual coinfections. Three of them were shown to have triple infections by ddPCR. In only one case, qPCR revealed a double infection in which ddPCR failed to detect it. OaPV coinfections by triple and quadruple genotypes were detected in 24 (~ 42.8%) and only one (~ 1.8%) of 56 multiple infections, respectively. Multiple infections caused by OaPV1/OaPV3/OaPV4 genotypes were the most prevalent ones being seen in 12 (50%) blood samples harboring triple OaPV infections. Neither triple nor quadruple infection was observed by real-time qPCR. Table 3 summarizes the coinfection results.

**Table 3:** Genotype coinfections by ddPCR with related number of their combination are shown.

**Coinfections**	**Genotype combination**	**Number**
Double	OaPV1/OaPV2	4
	OaPV1/OaPV3	3+1^†^
	OaPV1/OaPV4	5
	OaPV2/OaPV3	4
	OaPV2/OaPV4	3
	OaPV3/OaPV4	11
Triple	OaPV1/OaPV2/OaPV3	6
	OaPV1/OaPV2/OaPV4	4
	OaPV1/OaPV3/OaPV4	12
	OaPV2/OaPV3/OaPav4	2
Quadruple	OaPV1/OaPV2/OaPV3/OaPV4	1

† *An additional dual infection composed of OaPV1/OaPV3 genotype combination was detected by qPCR only*.

The overall quantification results showed that viral copy number/μL ranged from 0.22 to 207 for OaPV1, 0.17–2.85 for OaPV2, 0.18–4.98 for OaPV3, and 0.28–12.72 for OaPV4. In samples positive for both assays, the copy number of ddPCR was correlated with the Ct of real-time qPCR because the higher the copy number, the lower was the Ct of qPCR. The detailed results are summarized in Supplemental Table S1.

## Discussion

To the best of our knowledge, this study is the first systematic research on the molecular epidemiology of OaPV infection among sheep using real-time qPCR and ddPCR as diagnostic procedures. DdPCR revealed the nucleic acid of ovine *Delta*-PV (OaPV1, OaPV2, OaPV4), and *Dyokappa-*PV (OaPV3) in a very high percentage as it was able to detect OaPV DNA in 124 out of 126 positive blood samples (~ 98.4%). Our findings showed that ddPCR, which has not yet been utilized for studying OaPV epidemiology, is an advanced technology that can accurately diagnose OaPV infection with high specificity and sensitivity thus representing a promising new tool for the accurate detection and quantification of the OaPV load. qPCR failed to detect OaPV DNA in a large number of samples which, in contrast, harbored OaPV DNA, as detected *via* ddPCR, thus suggesting that DNA levels may be too low and traditional methods such as real-time qPCR may be faulty to detect them *via* blood samples. Therefore, this study demonstrated that ddPCR outperforms qRT-PCR in terms of sensitivity and specificity for OaPV detection.

We showed that OaPV4 and OaPV3 are the most prevalent OaPVs in sheep flocks in southern Italy, respectively. It is worth noting that OaPV3 and OaPV4 have been identified in tumors of sheep from the Mediterranean area only (16, 17), whereas OaPV1 and OaPV2 have not previously been reported in Italy. OaPV3 and OaPV4 achieved an overall higher viral load than OaPV1 and OaPV2. DdPCR assay showed a very high sensitivity as the LoD showed values < 1 copies/μL, which are believed to be a robust marker of the high sensitivity of the ddPCR protocol for research on virus, including PVs (27, 28). Furthermore, our study showed that diagnostic testing plays a critical role in addressing OaPV epidemiology and confirmed that qPCR is extremely inaccurate for detecting pathogens at very low concentrations, as previously suggested (29). A higher percentage of OaPV-positive samples detected by ddPCR showed that this assay offers the potential to perform precise low-level quantification otherwise undetectable thus allowing us to assess the epidemiology profile of OaPVs and gather insights into their territorial prevalence. In this context, our study confirmed that ddPCR can be used for low-abundance nucleic acid detection and is very useful in diagnosing infectious diseases, including viral infections in comparative medicine (29). In addition, ddPCR is very accurate and sensitive diagnostic assay for the detection and quantification of human papillomavirus DNA (25, 30, 31) and BPV DNA (22–24).

DdPCR testing is pivotal for accurate viral load measurements, OaPV epidemiological interpretations, and the health management of sheep flocks. Quantification of viral load may be very useful both as a diagnostic procedure and as a prognostic biomarker. Although the correlation between viral load and PV infection remains to be elucidated (32), it is believed that PV viral load is an important determinant of viral persistence (33). Furthermore, ddPCR significantly reduced the false negative rates of OaPV detection, which may be responsible for virus spread. It could be of epidemiological importance to know whether sheep harboring OaPVs, particularly those belonging to the *Delta*-PV genus, can represent a potential reservoir for intra- and inter-species transmission similar to cattle for bovine *Delta*-PV. Preliminary results of an ongoing study on the detection and quantification of OaPV DNA in the blood of cattle and goats confirm that OaPVs are characterized by cross-species transmission (Roperto, personal observations). In addition, OaPV2 DNA sequences have recently been found in a gingival sarcoid-like mass of a pig; therefore cross-species transmission of OaPVs may be possible (20). Both bovine and ovine *Delta*-PVs are characterized by overlapping biological properties, including cell tropism and pathogenicity (17, 34).

As OaPVs have been detected in healthy sheep, it is conceivable that blood represents an important primary route of infection; therefore, OaPVs can disseminate to any organs *via* the bloodstream. Epidemiological data on the territorial genotype prevalence of OaPVs are of interest as PV diseases appear to be associated with specific genotypes both in humans (35) and farm animals (5, 6, 36).

Finally, the high prevalence of OaPVs reported in the current study may represent an important, yet unknown threat to ovine industries. The improvement of virus detection in livestock remains a priority in clinical practice. This study showed that accurate diagnostic methods play a crucial role in OaPV control strategies. Therefore, ddPCR may represent the diagnostic molecular tool of choice, which may ultimately provide useful insights into molecular epidemiology and field surveillance, known to be key components of the control program of any infectious disease, including viral diseases. Further studies to better understand the risks posed by the infectivity of OaPVs and manage the potential clinical impact of PV-related diseases in sheep flocks are warranted.

## Data Availability Statement

The datasets presented in this study can be found in online repositories. The names of the repository/repositories and accession number(s) can be found in the article/Supplementary Material.

## Author Contributions

SR designed the experiments and wrote the manuscript. FD and AC carried out the experiments. PC, CC, and ND'A analyzed data. All authors read and approved the final manuscript.

## Conflict of Interest

The authors declare that the research was conducted in the absence of any commercial or financial relationships that could be construed as a potential conflict of interest.

## Publisher's Note

All claims expressed in this article are solely those of the authors and do not necessarily represent those of their affiliated organizations, or those of the publisher, the editors and the reviewers. Any product that may be evaluated in this article, or claim that may be made by its manufacturer, is not guaranteed or endorsed by the publisher.

## Footnotes

^1^Papillomavirus Episteme (PAVE) (2021). Available online at: http://pave.niaid.nih.gov/ (accessed September 20, 2021).

^2^https://eu.idtdna.com/scitools/Applications/RealTimePCR/ (accessed September 10, 2021).
