# Supplementary material for: Molecular Epidemiology of Ovine Papillomavirus Infections Among Sheep in Southern Italy
Source: Front Vet Sci. 2021 Nov 22;8:790392. doi: 10.3389/fvets.2021.790392 (PMC8645557; doi:10.3389/fvets.2021.790392)
Supplement: Supplementary file 1 [file Table_1.docx]

| **samples** | **OaPV1 ddPCR** | **OaPV1 qPCR** | **OaPV2 ddPCR** | **OaPV2 qPCR** | **OaPV3 ddPCR** | **OaPV3 qPCR** | **OaPV4 ddPCR** | **OaPV4 qPCR** |
| --- | --- | --- | --- | --- | --- | --- | --- | --- |
| P1 | N | N | N | N | N | N | N | N |
| P2 | N | N | N | N | N | N | 0.87 | N |
| P3 | N | N | N | N | N | N | 2.21 | N |
| P4 | N | N | N | N | 0.27 | N | N | N |
| P5 | N | N | N | N | 0.18 | N | 0.3 | N |
| P6 | N | N | N | N | 4.02 | 30.95 | N | N |
| P7 | N | N | N | N | N | N | 0.48 | N |
| P8 | N | N | N | N | N | N | 0.36 | N |
| P9 | N | N | N | N | 0.26 | 36.3 | N | N |
| P10 | 1.87 | N | N | N | N | N | N | N |
| P11 | N | N | N | N | N | N | N | N |
| P12 | N | N | N | N | 1.1 | 32.55 | N | N |
| P13 | N | N | N | N | N | N | 0.31 | N |
| P14 | N | N | N | N | 1.2 | 32.8 | N | N |
| P15 | N | N | N | N | 0.57 | N | N | N |
| P16 | N | N | N | N | 0.34 | N | N | N |
| P17 | N | N | 1.48 | N | 2.44 | N | N | N |
| P18 | N | N | N | N | 0.69 | 39.48 | N | N |
| P19 | N | N | N | N | 2.1 | N | N | N |
| P20 | N | N | N | N | 1.55 | N | N | N |
| P21 | 1.1 | N | 0.3 | 39.63 | 4.68 | 30.15 | N | N |
| P22 | 0.77 | N | N | N | N | 38.95 | N | N |
| P23 | N | N | N | N | N | N | N | N |
| P24 | 0.43 | N | N | N | 0.8 | 36.1 | N | N |
| P25 | N | N | N | N | N | 39.2 | 0.7 | N |
| P26 | N | N | N | N | 1.73 | 33.89 | 1.64 | N |
| P27 | N | N | N | N | N | N | N | N |
| P28 | 0.81 | N | 0.311 | N | 3.86 | 31.2 | N | N |
| P29 | N | N | 2.16 | N | 2.01 | 34.73 | 0.74 | N |
| P30 | N | N | N | N | 1.28 | 32.7 | N | N |
| P31 | N | N | N | N | 0.36 | N | 1.57 | N |
| P32 | 2.79 | N | N | N | N | N | 0.42 | N |
| P33 | 0.87 | N | N | N | N | N | N | N |
| P34 | N | N | N | N | N | N | 0.47 | N |
| P35 | N | N | N | N | N | N | 0.36 | N |
| P36 | N | N | N | N | 1.32 | 31.9 | 0.37 | N |
| P37 | N | 37.82 | N | N | 1.17 | 31.35 | N | N |
| P38 | 0.22 | N | 1.2 | N | 1.82 | 31.09 | N | N |
| P39 | 0.35 | N | N | N | 2.26 | 33.8 | 0.38 | N |
| P40 | 1.59 | N | N | N | 3.52 | 30.37 | N | N |
| P41 | 1.11 | N | 0.27 | N | N | N | 0.65 | N |
| P42 | N | N | N | N | 0.31 | N | 0.83 | N |
| P43 | N | N | N | N | N | N | N | N |
| P44 | 0.86 | N | 2.71 | N | 3.23 | 30.2 | 0.61 | N |
| P45 | 207 | 16.06 | N | N | 1.3 | 33.05 | 0.38 | N |
| P46 | 0.53 | N | N | N | 0.65 | N | 1.05 | 34.62 |
| P47 | 0.5 | N | 0.5 | N | N | N | 1.6 | 34.46 |
| P48 | N | N | N | N | 1.77 | N | 2.41 | 33.98 |
| P49 | 0.24 | N | N | N | 0.94 | N | 0.59 | 39.7 |
| P50 | N | N | 0.59 | N | N | N | N | N |
| P51 | N | N | N | N | N | N | 1.01 | 34.17 |
| **samples** | **OaPV1 ddPCR** | **OaPV1 qPCR** | **OaPV2 ddPCR** | **OaPV2 qPCR** | **OaPV3 ddPCR** | **OaPV3 qPCR** | **OaPV4 ddPCR** | **OaPV4 qPCR** |
| P52 | N | N | N | N | 0.71 | N | N | N |
| P53 | N | N | 0.28 | N | 0.53 | N | 2.3 | 33.5 |
| P54 | N | N | N | N | N | N | 1.11 | N |
| P55 | N | N | N | N | N | N | N | N |
| P56 | 0.72 | N | 0.26 | N | N | N | N | N |
| P57 | 0.22 | N | N | N | 0.89 | N | 0.4 | N |
| P58 | 0.23 | N | N | N | N | N | N | N |
| P59 | 0.23 | N | N | N | 0.33 | N | 1.6 | 34.58 |
| P60 | N | N | 0.17 | N | N | N | N | 37.19 |
| P61 | 0.23 | N | 0.25 | N | N | N | N | N |
| P62 | N | N | 0.27 | N | N | N | N | N |
| P63 | N | N | 1 | 35.63 | N | N | N | N |
| P64 | N | N | N | N | N | N | 1.04 | 34.03 |
| P65 | N | N | N | N | N | N | N | N |
| P66 | 0.29 | N | N | N | 0.99 | N | N | N |
| P67 | 0.24 | N | N | N | N | N | N | N |
| P68 | N | N | N | N | 0.53 | N | 0.66 | N |
| P69 | N | N | N | N | N | N | N | N |
| P70 | N | N | N | N | N | N | 1.33 | N |
| P71 | 0.23 | N | N | N | 0.27 | N | 0.36 | N |
| P72 | 0.26 | N | N | N | 0.74 | N | 2.4 | 33.4 |
| P73 | N | N | N | N | N | N | N | N |
| P74 | N | N | N | N | N | N | 12.72 | N |
| P75 | N | N | N | N | N | N | N | N |
| P76 | N | N | 0.21 | N | 0.64 | N | N | N |
| P77 | 0.23 | N | 0.44 | N | 1.26 | N | N | N |
| P78 | N | N | 0.24 | N | 1.73 | N | 1.73 | 34.23 |
| P79 | 0.25 | N | N | N | N | N | 1.64 | N |
| P80 | N | N | N | N | N | N | N | N |
| P81 | 0.25 | N | N | N | N | N | N | N |
| P82 | N | N | 0.52 | N | N | N | N | N |
| P83 | 0.24 | N | N | N | N | N | N | N |
| P84 | N | N | N | N | N | N | N | N |
| P85 | N | N | N | N | 2.2 | 33.34 | 0.93 | 37.34 |
| P86 | N | N | N | N | N | N | N | N |
| P87 | N | N | 0.46 | N | 0.72 | N | N | N |
| P88 | N | N | N | N | N | N | N | N |
| p89 | N | N | N | N | N | N | 2.04 | 34.95 |
| p90 | N | N | N | N | N | N | 0.96 | N |
| p91 | N | N | 0.52 | N | N | N | N | N |
| p92 | N | N | N | N | N | N | N | N |
| p93 | N | N | 0.23 | N | 0.4 | N | N | N |
| p94 | N | N | N | N | N | N | 1.98 | N |
| p95 | N | N | 0.23 | N | N | N | N | N |
| p96 | N | N | N | N | N | N | N | N |
| p97 | N | N | N | N | 0.39 | N | 0.65 | N |
| p98 | N | N | N | N | N | N | N | N |
| p99 | N | N | 0.69 | N | N | N | N | N |
| p100 | N | N | N | N | N | N | N | N |
| p101 | 0.48 | N | 0.47 | 39.87 | N | N | 1.1 | N |
| p102 | N | N | 0.24 | 39.94 | N | N | N | N |
| **samples** | **OaPV1 ddPCR** | **OaPV1 qPCR** | **OaPV2 ddPCR** | **OaPV2 qPCR** | **OaPV3 ddPCR** | **OaPV3 qPCR** | **OaPV4 ddPCR** | **OaPV4 qPCR** |
| p103 | N | N | N | N | N | N | 5.8 | N |
| P104 | N | N | N | N | N | N | 0.38 | N |
| P105 | 0.26 | N | 2.85 | N | N | N | 0.28 | N |
| P106 | 0.24 | N | N | N | N | N | N | N |
| P107 | N | N | N | N | 0.3 | N | N | N |
| P108 | N | N | 0.27 | N | N | N | N | N |
| P109 | N | N | N | N | 2.04 | 33.68 | N | N |
| P110 | N | N | N | N | N | N | N | N |
| P111 | N | N | N | N | N | N | N | N |
| P112 | N | N | N | N | 2.5 | 33.28 | 0.3 | N |
| P113 | N | N | N | N | N | N | N | N |
| P114 | N | N | N | N | N | N | 1.51 | N |
| P115 | N | N | N | N | N | N | N | N |
| P116 | N | N | N | N | N | N | N | N |
| P117 | 0.29 | N | 0.27 | N | N | N | 0.56 | N |
| P118 | N | N | N | N | N | N | N | N |
| P119 | N | N | N | N | 0.56 | N | 1.5 | 34.26 |
| P120 | N | N | N | N | 1.63 | 32.18 | N | N |
| P121 | N | N | N | N | 4.98 | 31.25 | N | N |
| P122 | N | N | N | N | N | N | N | N |
| P123 | N | N | N | N | N | N | N | N |
| p124 | N | N | 0.22 | N | N | N | N | N |
| p125 | N | N | N | N | N | N | N | N |
| p126 | 0.26 | N | N | N | 0.26 | N | 0.45 | N |
| p127 | N | N | N | N | N | N | N | N |
| p128 | N | N | N | N | N | N | N | N |
| p129 | N | N | N | N | N | N | N | N |
| p130 | N | N | N | N | 0.31 | N | 0.63 | N |
| p131 | 0.24 | N | N | N | N | N | 0.36 | N |
| p132 | N | N | N | N | N | N | N | N |
| p133 | N | N | N | N | N | N | 0.36 | N |
| p134 | 0.24 | N | N | N | N | N | N | N |
| p135 | N | N | 1.1 | N | N | N | N | N |
| p136 | N | N | N | N | N | N | 0.33 | N |
| p137 | N | N | N | N | N | N | N | N |
| p138 | N | N | N | N | N | N | 0.28 | N |
| p139 | 3.58 | N | N | N | N | N | N | N |
| p140 | N | N | N | N | N | N | N | N |
| p141 | N | N | 0.26 | N | N | N | N | N |
| p142 | 2.69 | N | 0.24 | N | N | N | 0.36 | N |
| p143 | N | N | N | N | N | N | 0.58 | N |
| p144 | N | N | N | N | N | N | N | N |
| p145 | N | N | N | N | 0.33 | N | N | N |
| p146 | N | N | N | N | 0.39 | N | 0.68 | N |
| p147 | N | N | N | N | 0.34 | N | N | N |
| p148 | N | N | N | N | N | N | N | N |
| p149 | N | N | 0.4 | N | N | N | N | N |
| p150 | 0.54 | N | 0.48 | N | 0.3 | N | N | N |
| p151 | N | N | N | N | N | N | N | N |
| p152 | N | N | N | N | N | N | N | N |
| p153 | 0.6 | N | 0.25 | 38.65 | N | N | N | N |
| **samples** | **OaPV1 ddPCR** | **OaPV1 qPCR** | **OaPV2 ddPCR** | **OaPV2 qPCR** | **OaPV3 ddPCR** | **OaPV3 qPCR** | **OaPV4 ddPCR** | **OaPV4 qPCR** |
| p154 | 2.9 | N | 0.41 | N | N | N | N | N |
| p155 | N | N | N | N | N | N | N | N |
| p156 | N | N | N | N | N | N | N | N |
| p157 | 0.75 | N | N | N | N | N | N | N |
| p158 | 0.24 | N | N | N | 0.31 | N | 0.5 | N |
| p159 | 2.58 | 34.41 | N | N | N | N | N | N |
| p160 | N | N | N | N | N | N | 0.56 | N |
| p161 | 0.05 | N | N | N | N | N | 0.97 | N |
| p162 | 0.25 | N | N | N | N | N | 0.34 | N |
| p163 | N | N | N | 32.18 | 0.3 | N | 0.36 | N |
| p164 | N | N | N | 30.06 | N | N | N | N |
| p165 | N | N | N | N | N | N | 0.36 | N |

**Supplemental Table S1** - Overall results of droplet digital polymerase chain reaction (ddPCR), expressed as number of copies/μL, and of real-time quantitative PCR (qPCR), expressed as threshold cycle (Ct), for all 165 samples, N = ovine papillomavirus (OaPV) DNA not detected.
